# A Positive Causal Relationship between Noodle Intake and Metabolic Syndrome: A Two-Sample Mendelian Randomization Study

**DOI:** 10.3390/nu15092091

**Published:** 2023-04-26

**Authors:** Sunmin Park, Meiling Liu

**Affiliations:** 1 Obesity/Diabetes Research Center, Department of Food and Nutrition, Hoseo University, Asan 31499, Republic of Korea; meiling1125@naver.com; 2 Shanxi Institute of Science and Technology, College of Chemical Engineering, Jincheng 048011, China

**Keywords:** Mendelian randomization, noodle intake, diet quality, metabolic syndrome

## Abstract

The controversy over the link between noodle consumption and metabolic syndrome (MetS) persists. Using a two-sample Mendelian randomization (MR) approach, we aimed to examine the potential causal relationship between noodle consumption and the risk of MetS and its components in adult populations of city hospital-based (*n* = 58,701) and Ansan/Ansung plus rural (AAR; *n* = 13,598) cohorts. The instrumental variables were assigned with genetic variants associated with low- and high-noodle intake (cutoff: 130 g/day) by a genome-wide association study (GWAS) with *p* < 5 × 10^−5^ and linkage disequilibrium (r^2^ = 0.001), following adjustment for covariates related to MetS, in the city cohort. MR-Egger, inverse-variance weighted (IVW), and weighted median were applied to investigate the causal association of noodle intake with MetS risk in the AAR. The quality of the MR results was checked with leave-one-out sensitivity and heterogeneity analyses. A higher energy intake with lower carbohydrates and higher fats, proteins, and higher sodium and a lower intake of calcium, vitamin D, vitamin C, and flavonoids were shown in the high-noodle group, indicating poor diet quality. The glycemic index and glycemic load of daily meals were much higher in the high-noodle intake group than in the low-noodle intake group. In the observational studies, not only the total noodle intake but also the different types of noodle intake were also positively associated with MetS risk. In the MR analysis, high-noodle intake elevated MetS, hypertension, dyslipidemia, hyperglycemia, hypertriglyceridemia, and abdominal obesity in an IVW model (*p* < 0.05) but not the MR-Egger model. No single genetic variant among the instrumental variables changed their relationship in the leave-one-out sensitivity analysis. No likelihood of horizontal pleiotropy and heterogeneity was exhibited in the association between noodle intake and MetS. In conclusion, noddle intake had a positive causal association with MetS and its components in Asian adults.

## 1. Introduction

Metabolic syndrome (MetS) is a group of disease conditions that raise the risk of type 2 diabetes (T2DM), coronary heart disease, and stroke. A diagnosis of MetS is made when an individual has three or more of the following components: abdominal obesity, hypertension, hyperglycemia, hypo-HDL-cholesterolemia, and hypertriglyceridemia. MetS is a significant global public health issue, affecting approximately one-quarter of the world’s population [1]. The prevalence of MetS is higher in developed countries, with rates ranging from 20 to 30% in Europe and North America to over 40% in India and China among the Asian countries and in Mexico in South America. Indeed, the incidence of MetS is rising rapidly in developing countries. In Korea, MetS increased among men from 24.5% to 28.1% during the decade 2008–2017. However, it was 20.5% among women in 2008 and 18.7% in 2017 [2].

The incidence of MetS differs between locations depending on environmental factors, although genetics can interact with environmental factors to affect the probability of developing MetS. The rapid increase in MetS incidence in developing countries has been linked to a shift toward more sedentary lifestyles and the higher consumption of high-calorie and fat-rich diets that increase insulin resistance [3]. A healthy diet full of fruits, vegetables, whole grains, nuts, legumes, and fish, and with low levels of saturated fats, trans fats, and added sugars, has been linked to a reduced risk of MetS. These healthy dietary patterns include the Mediterranean diet, Dietary Approaches to Stop Hypertension (DASH), and Korean-balanced diets [4]. These dietary patterns have been reported to have an inverse relationship with MetS risk [5]. The shift from a Korean-balanced diet to a Western-style diet (WSD) increases MetS incidence, and a WSD is positively associated with MetS risk [5]. However, Koreans have not consumed meat as much as people in Western countries (men: 96 g/day and women: 76 g/day), and meat intake does not differ between non-MetS and MetS groups in Korea [6,7]. Interestingly, the WSD among Koreans is characterized by a high intake of noodles consumed with meat and processed meats. However, since the intake of meats and processed meats remains lower in Korea than in Western countries, a high carbohydrate diet (about 70 percent energy) and not a high-fat diet strongly correlates with MetS risk in Korea [8]. Therefore, a higher intake of noodles rich in carbohydrates may be linked to MetS risk. 

The association of noodle (or pasta) intake on MetS risk has been evaluated in previous studies. Noodles are made not only of refined flour but also rice and buckwheat. However, noodle dishes are high carbohydrate and calorie-rich foods. In an earlier study conducted on the data from the Korean National Health and Nutrition Examination Survey (KNHANES) IV 2007–2009, increased intake of instant noodles (≥2 times/week) was positively associated with MetS risk by 1.68-fold in women [7]. In addition, the higher intake of instant noodles (≥3 times/week) was positively linked to a 2.6-fold higher risk of dyslipidemia among Korean college students [9]. However, the effects of other types of noodles or pasta on MetS risk in Korea remain unknown. In a systematic review and meta-analysis of thirteen randomized clinical trials conducted in Western countries, the intake of pasta was found to have significantly lower standard mean differences in postprandial glucose responses compared to bread (seven studies) or potatoes (six studies) [10]. However, the intake of pasta made from whole grains has not shown beneficial effects on glucose metabolism compared to that made from refined grains [10]. Furthermore, pasta meals substituted for bread or potatoes were inversely associated with stroke and cardiovascular disease in the Women’s Health Initiative (WHI), a series of clinical studies initiated by the US National Institutes of Health (NIH). However, pasta intake did not show a significant association with T2DM risk [10]. Moreover, pasta consumption had an inverse relationship with the risk of being overweight and obese in an Italian Nutrition & Health Survey (INHES) [11]. Therefore, the effects of pasta consumption on MetS risk remain controversial. 

The present study aimed to determine the effects of consuming different types of noodles on MetS risk and its components. Their causal relationship was studied by carrying out a two-sample Mendelian randomization (MR) analysis on adults aged over 40 in the Korean Genomic Epidemiology Study (KoGES), a long-term population-based cohort study. It is novel to demonstrate that different types of noodle intake were similarly associated with the risk of MetS and its components and to show the causal association of overall noodle consumption with the risk of MetS and its components by randomizing participants with genetic variants related to the noodle consumption.

## 2. Methods

### 2.1. Participants

Between 2004 and 2013, the Korea Centers for Disease Control and Prevention (KCDC) established the KoGES, which contained three cohorts—rural (*n* = 8105), Ansan/Ansung (*n* = 5493), and hospital-based urban (*n* = 58,701). The KoGES obtained approval from the KoGES Institutional Review Board (KBP-2019-055), and this study was approved by Hoseo University Institutional Review Board (1041231-150811-HR-034-01) to comply with the Declaration of Helsinki. All the participants agreed to the terms after providing written informed consent.

### 2.2. Demographic, Anthropometric, Biochemical, and Clinical Parameter Assessment

At the first visit, the participants completed the survey questionnaire providing data such as their gender, age, monthly income (<$2000, $2000–4000, or >$4000), education (<high school, high school, or college plus above), type, amount, and frequency of alcohol consumption, smoking status, and physical activity [12,13]. The daily alcohol intake (g/day) was calculated by multiplying the alcohol percentage according to alcohol drink types and amount on a single occasion by the frequency of consumption. It was categorized into low (0–19 g) and high drinkers (≥20 g) [14]. Current and past smokers were divided based on smoking over 100 cigarettes during the six months prior to the survey in their lifetimes [13]. 

According to a standardized procedure, anthropometric measurements were taken, with the participants wearing light clothes and barefoot [15]. Blood was drawn from the participants after they had fasted for over 12 h, with and without anticoagulants. The plasma and serum were separated from the blood after it was centrifuged at 500× *g*. Plasma glucose, serum lipid profiles, serum alanine transaminase (ALT), serum aspartate transaminase (AST), and serum creatinine concentrations were analyzed using an automatic analyzer (Hitachi 7600, Hitachi, Tokyo, Japan) [12]. The glycated hemoglobin (hemoglobin A1c; HbA1c) levels were measured using ethylenediaminetetraacetic acid (EDTA)-treated blood, and serum high-sensitive C-reactive protein (hs-CRP) concentrations were assessed with an enzyme-linked immunosorbent assay (ELISA) kit (R&D system, Minneapolis, MN, USA). The estimated glomerular filtration rate (eGFR) was calculated using MDRD Study Equation [16]. Blood pressure was taken three times from the right arm in a sitting position while the participant was sitting, and the averages of SBP and DBP were used. 

### 2.3. MetS Definition

MetS is a cluster of conditions that includes a minimum of three of the following criteria, according to the International Diabetes Federation (IDF) and the criteria for MetS of the Korean Society for the Study of Obesity (KSSO): abdominal obesity (90 cm for men and 85 cm for women), hypertension (average systolic blood pressure ≥ 130 mmHg or diastolic blood pressure ≥ 85 mmHg or currently on antihypertensive medication), hyperglycemia (≥100 mg/dL or currently on the hypoglycemic agent), hypertriglyceridemia (≥150 mg/dL or currently on anti-dyslipidemic medication), and hypo-HDL-cholesterolemia (<40 mg/dL for men, and <50 mg/dL for women or currently on anti-dyslipidemic medication) [17]. MetS can be considered a borderline or a “pre”-condition between healthy and disturbed metabolisms, which could progress to specific metabolic diseases such as T2DM.

### 2.4. Food and Nutrient Intake Measurement

The semi-quantitative food frequency questionnaire (SQFFQ), fitted to the eating habits of Koreans, has been developed and validated for the KoGES [18] and was completed based on the food consumed within the 6-month period prior to the survey. According to the SQFFQ results, the daily intake of 106 foods was assessed from the frequencies and amounts of each food. The intake of noodles was divided into five categories, namely, instant noodles, Chinese noodles (Chajangmyon/Jambbong), buckwheat noodles, wheat noodles, and starch noodles. The total noodle intake was calculated by summing the noodle intake of the five categories. The nutrients present in the results were calculated based on the food intake from the SQFFQ using the computer-aided nutrition analysis program (CAN-Pro) 3.0, a nutrient database program developed by the Korean Nutrition Society (Seoul, Republic of Korea) [18]. 

### 2.5. Dietary Patterns, Dietary Inflammatory Index (DII), Glycemic Index (GI), and Glycemic Load (GL)

Foods in the SQFFQ were categorized into 29 food groups according to nutrient similarity. Dietary patterns were chosen according to an eigenvalue > 1.5 by performing the principal component analysis (PCA) [18]. The predominant food groups in each pattern were selected with ≥0.40 factor-loading values after applying the orthogonal rotation procedure [15]. The names of each dietary pattern were based on the primary food group chosen (Supplementary Table S1).

The dietary inflammatory index (DII) of each participant was determined by multiplying the inflammatory weights of the following four groups: energy sources, thirty-two nutrients, caffeine, and four spices by the intake of each participant and divided by 100 [19].

The GI and GL were calculated with the corresponding equations. The GI of the same food can vary due to differences in the types of the food and its nutritional composition. Hence the GI values commonly listed for Korean foods were used [20,21]. The GI and GL of daily food intake in the form of meals were calculated by following equations [22]:

GI of daily food intake = $\sum \begin{array}{c} i=n\\ i=1 \end{array}$(GI of a food X carbohydrate in the food)*i*/total carbohydrate intake for a day.

GL of daily food intake = $\sum \begin{array}{c} i=n\\ i=1 \end{array}$(GI of a food X carbohydrate in the food)*i*/100

*n*: the number of foods consumed in a day.

### 2.6. Genotyping and Quality Control

The participants’ genotypes were assessed at the Center for Genome Science of the Korea National Institute of Health (Osong, Republic of Korea). The resulting data were then provided to the researchers. Genomic DNA extracted from the whole blood was used to perform genotyping using a Korean chip (Affymetrix, Santa Clara, CA, USA) that was specifically designed to look for genetic variants linked to chronic metabolic diseases (dyslipidemia, diabetes, hypertension, osteoporosis, kidney disease, and autoimmune diseases) and cancers in Korean adults [15,23]. The genotyping accuracy was verified by the Bayesian Robust Linear Model (BRLMM) genotyping algorithm [24]. The quality of the genotyping was further checked with the following criteria: absence of gender bias, low heterozygosity (<30%), genotyping accuracy (≥98%), missing genotype call rates (<4%), minor alleles frequency (MAF, >1%), and Hardy–Weinberg Equilibrium (HWE; *p* > 0.05) [23]. After genotype imputation, the genotype quality was then verified with additional inclusion criteria of a posterior probability score > 0.90 and low informative content of genotype informative > 0.5.

### 2.7. Identification of Instrumental Variables in a Two-Sample MR Analysis

A genome-wide association study (GWAS) was conducted to investigate the genetic variants in relation to the daily total noodle intake. The daily total noodle intake was divided into two categories with a cutoff of 130 g cooked noodles per day (3 times/week): low-noodle (<130 g/d) and high-noodle (≥130 g/d) [14,25]. This study was performed utilizing PLINK v.2.0 (http://pngu.mgh.harvard.edu/~purcell/plink accessed on 12 May 2022) in a city hospital-based cohort (*n* = 58,701). The following factors were considered as covariates: participants’ age, residential area, gender, body mass index (BMI), education, daily physical activity, energy intake, carbohydrate intake, alcohol consumption, and smoking status. The *p*-values of the genetic variants from the association between the low- and high-noodle groups were plotted with the GWAS. The *p*-values of the genetic variants were then plotted in a Manhattan plot to distinguish significant genetic variants on chromosome 12 (Supplementary Figure S1A). The quantile–quantile (Q-Q) plot of the observed and expected *p*-values revealed only a few sources of spurious associations (Supplementary Figure S1B). The genome inflation factor (λGC) value in the Q-Q plot was estimated to be 1.016, indicating that the genetic variants from the GWAS did not have large inflation and an excessive false positive rate. The gene name of the SNP was identified by searching the g: profiler database website (https://biit.cs.ut.ee/gprofiler/snpense, accessed on 3 June 2022).

The linkage disequilibrium (LD) of all selected genetic variants within a 10,000 kb distance was checked: the variants were considered for inclusion if their r2 value was less than 0.001 (or D’ < 0.2) in the R program. The significance of the genetic variants influencing noodle intake was liberally set to *p* < 5 × 10^−5^ as the majority of the variants (over 50%) showed linkage disequilibrium and a large number of participants (*n* = 58,701) were included in the study. Previous studies also used a liberal significance level, especially where lifestyles were instrumental variables [26]. Supplementary Table S2 presents potential instrumental variables related to noodle intake, with the respective gene names sourced from the g:profiler database website (https://biit.cs.ut.ee/gprofiler/snpense, accessed on 18 June 2022).

### 2.8. A Two-Sample MR Analysis Design

**Figure 1:**
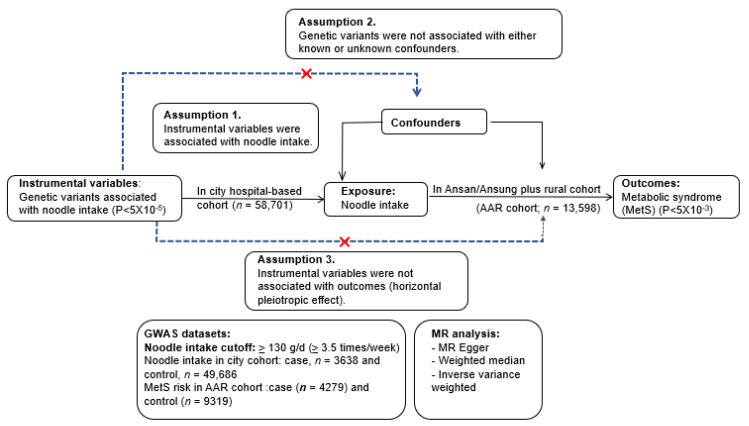
Experimental flow and assumptions for a two-sample Mendelian randomization (MR) of the causal association of daily total noodle intake with the risk of metabolic syndrome and its components.

In the noodle intake (exposure) and MetS and its components (outcomes), the genetic variants (instrumental variables) that affect noodle intake were used to randomize the participants to evaluate the association between noodle intake and MetS and its components. Figure 1 displays the diagram of the two-sample MR design. The results of the two-sample MR analysis can show the causal relationship between the noodle intake and MetS and its components when the assumptions are fulfilled. The assumptions were as follows: (1) The genetic variants should be connected to the noodle intake and serve as instrumental variables; (2) The instrumental variables must not be associated with confounders; (3) The instrumental variables should have no effect on the risk of MetS and its components [25]. The confounders included categorical variables (gender, residential area, education, income, exercise, and smoking) and continuous variables (age, BMI, and energy, carbohydrates, and alcohol intake). The assumptions were tested at a statistical significance level at *p* < 5 × 10^−5^, the same as the significance level for the instrumental variables. The assumptions in this study achieved the required statistical significance.

A two-sample MR analysis was conducted with the Mendelian Randomization package (v.0.5.1) and Two-Sample MR (v.0.4.26) in the R program. The scheme of a two-sample MR analysis is depicted in Figure 1. The KoGES cohorts were separated into two cohorts in order to satisfy the two-sample MR assumptions: (1) A city hospital-based cohort (*n* = 58,701) and (2) Ansan/Ansung plus rural cohorts (*n* = 13,598). Instrumental variables were created from the GWAS between individuals with low- and high-noodle consumption in the city hospital-based cohort (low-noodle: *n* = 52,408; high-noodle: *n* = 4493), as mentioned above. The association of instrumental variables with MetS and its components was conducted by logistic regression analysis in the Ansan/Ansung plus rural cohorts (*n* = 13,598). If the genetic variants selected for instrumental variables were linked to T2DM at *p* < 5 × 10^−5^ in the Ansan/Ansung plus rural cohorts, they were taken out of the instrumental variables. The number of participants in MetS and its metabolic traits in the Ansan/Ansung plus rural cohorts (*n* = 13,598) were (1) MetS (*n* = 4279); (2) abdominal obesity determined by waist circumferences (*n* = 4144); (3) hyperglycemia (*n* = 999); (4) hypo-HDL-cholesterolemia (*n* = 6277); (5) hypertriglyceridemia (*n* = 2306); and (6) hypertension (*n* = 4355).

The correlation between the amount of noodles consumed daily and the risk of metabolic syndrome and its components was calculated using a two-sample MR. The figure outlines the criteria of the instrumental variables as well as the assumptions for the MR analysis. All three assumptions of the MR analysis were fulfilled in this study.

The causal relationship between noodle intake and MetS incidence was determined with the two-sample MR analysis using the Mendelian Randomization package (v.0.5.1) and Two-Sample MR (v.0.4.26) in the R program. The MR package included the inverse-variance weighted (IVW) method, MR-Egger, weighted median, and weighted mode. IVW is the primary causal effect estimate with a robust ability to assume that all genetic variants are effective instrumental variables [27,28]. However, it requires exposure to the genetic variants (instrumental variables) that affect the target outcome only. Furthermore, other unknown confounding factors may contribute to horizontal pleiotropy and bias in effect size estimates, although the confounding factors that affect exposure were mostly excluded [27,28]. MR-Egger, IVW, and weighted median approaches were used to analyze the causal relationship of noodle intake to MetS and its components in the Ansan/Ansung plus rural cohorts (*n* = 13,598).

The heterogeneity test was used mainly to examine the differences between the selected IVs. The selected IVs were inappropriate if there was a strong heterogeneity between IVs with a small *p* value. The heterogeneity should not exist in IVs to use for MR study. The leave-one-out sensitivity test was used to conduct the MR with the remaining IVs after excluding IVs one by one. When the MR results estimated by removing one IV were very different from those with all IVs, the MR result was sensitive to excluding one IV. If one IV occupies a big portion of the MR results, the MR model is not robust. No matter which SNP is removed, the results are similar, indicating the MR result is robust. The analysis was included in the two-sample MR analysis.

### 2.9. Statistical Analysis

It was conducted using SAS v.9.3 (SAS Institute, Cary, NC, USA). The descriptive summaries for the categorical variables (e.g., gender, education, income, and smoking) were computed with frequency distribution according to the low- and high-noodle intake groups. The Chi-square test was utilized to determine the considerable variations in frequency distributions between the low- and high-noodle intake groups. After adjusting for the covariates, the adjusted means and standard deviations of the continuous variables were studied in the low- and high-noodle intake groups. Analysis of covariance (ANCOVA) was employed to assess the statistical discrepancies between the low- and high-noodle intake groups.

## 3. Results

### 3.1. Demographic Characteristics and Lifestyles of the Participants

The participants in the high-noodle group were, on average, younger than those in the low-noodle group (Table 1). A higher proportion of males was found within the high-noodle group than females (Table 1). Additionally, women with a higher education level and income were more likely to be in the high-noodle group than the low-noodle group (Table 1). Furthermore, the high-noodle group had a greater rate of alcohol consumption, less physical activity, and more smoking than the low-noodle group (Table 1).

### 3.2. Food and Nutrient Intake

The participants in the high-noodle group had a much higher energy intake than those in the low-noodle group, with the former consuming more energy than the estimated requirement (Table 1). Carbohydrates intake was lower in the high-noodle group, while fat and protein intake were higher (as shown in Table 1). Moreover, the former had a lower calcium, potassium, vitamin C, vitamin D, flavonoids, and cholesterol intake than the latter. However, sodium intake and DII were higher in the high-noodle group compared to the low-noodle group (Table 1).

The consumption of noodles among males in the high-noodle group was 4.6 times more than in the low-noodle group. The intake of noodles among females in the high-noodle group was 7.6 times more than among those in the low-noodle group (Table 2). The varieties of noodles were divided into instant noodles, wheat noodle soup, Chinese noodles, buckwheat noodles, and starch noodles, with intakes of all types except starch noodles significantly greater in the high-noodle group than in the low-noodle group (Table 2). The most regularly consumed noodles were wheat noodle soup and Chinese noodles.

GI and GL values of the daily meal were substantially higher for the high-noodle group than the low-noodle group for both genders, with men having higher values than women (Table 2). The high-noodle group participants had a higher intake of white rice but lower consumption of other foods such as whole grains, potatoes, vegetables, kimchi, fruits, beans, seaweed, meats, processed meats, fish, and nuts (Table 2).

The four dietary patterns were the Korean-balanced diet (KBD) for factor 1, the plant-based diet (PBD) for factor 2, the Western-style diet (WSD) for factor 3, and the rice-main diet (RMD) for factor 4. Regardless of dietary patterns, the proportion of noodle intake was higher in the high-noodle intake groups than in the low-noodle intake groups (Table 2). Interestingly, the proportion of noodle intake was 95.4% in men and 91.2% in women in WSD (Table 2), indicating that adults having WSD highly consumed noodles. 

### 3.3. Observational Association of Noodle Intake, MetS, and Its Components

**Figure 2:**
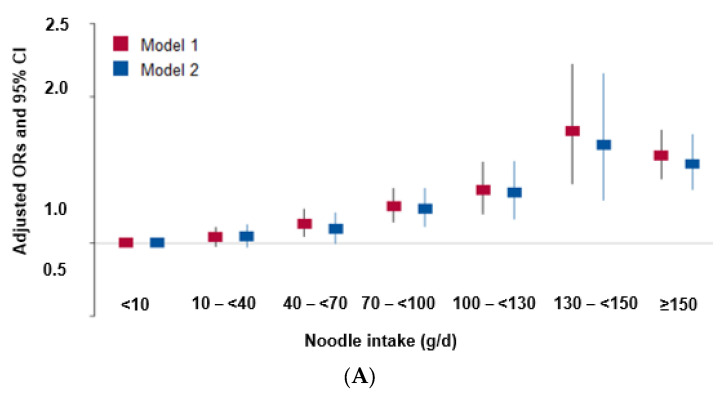

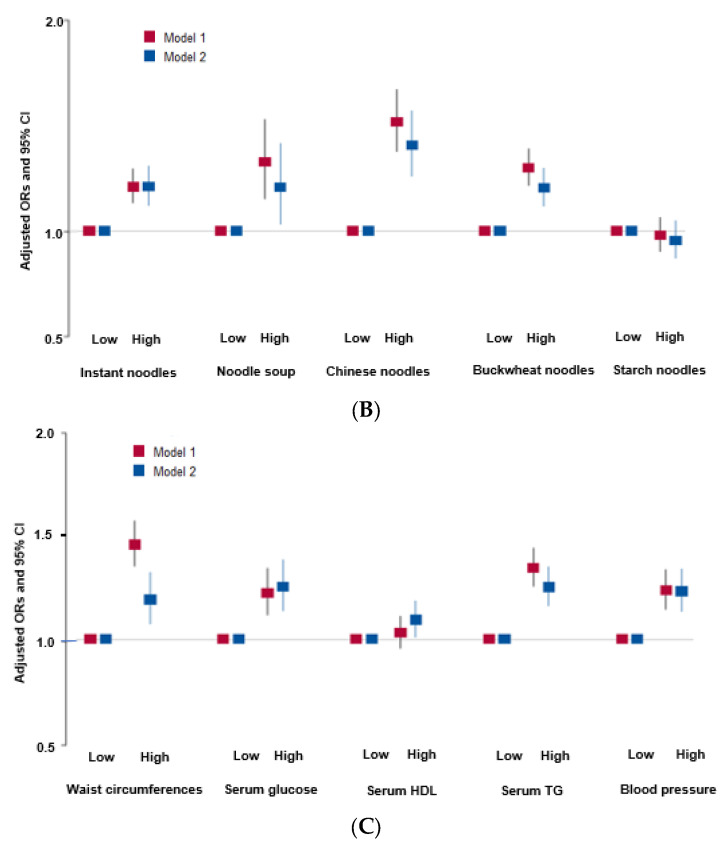
Association of noodle intake and its components with the risk of metabolic syndrome and its components in observational estimates in a city hospital-based cohort. (**A**) Association of the total noodle intakes by six groups (<10 g, 10–40 g, 40–70 g, 100–130 g, 130–150 g, ≥150 g total noodle intake/day) with metabolic syndrome and its components. Daily total noodle intake was calculated by summing up the daily intakes of instant noodles, noodle soup, Chinese noodles, buckwheat noodles, and starch noodles. (**B**) Association of the individual noodle intakes by two groups (cutoff of 130 g/day) with metabolic syndrome. The cutoffs of instant noodles, noodle soup, Chinese noodles, buckwheat noodles, and starch noodles were 25, 55, 40, 9, and 0.8 g/day, respectively. (**C**) Association of the daily total noodle intake by two groups (cutoff of 130 g/day) with metabolic syndrome components. Daily total noodle intake was calculated by summing the daily intakes of instant noodles, noodle soup, Chinese noodles, buckwheat noodles, and starch noodles.

After adjusting for two different sets of covariates, the total intake of noodles was positively associated with MetS risk in a dose-dependent manner (Figure 2A). The 130 g/day total noodle intake showed the highest adjusted ORs for MetS risk, and it was used as the cutoff. The association of each type of noodle with MetS is presented in Figure 2B, where the cutoff of 25 g, 55 g, 40 g, 9 g, and 0.8 g per day was assigned for instant noodles, noodle soup, Chinese noodles, buckwheat noodles, and starch noodles, respectively. Among the types of noodles, the intake of instant noodles, noodle soup, Chinese noodles, and buckwheat noodles was associated with MetS, but the intake of starch noodles was not. Participants with high total noodle intake (≥130 g/day) had a higher risk of MetS and its components compared with those with low-noodle intake in both genders after adjusting for the covariates related to MetS (Table 3). 

Among the MetS components, total noodle intake showed a positive relationship with abdominal obesity, hyperglycemia, hypertension, and hypertriglyceridemia in total participants. However, noodle intake had no association with hypo-HDL-cholesterolemia (Figure 2C). Table 3 also showed that the total noodle intake was positively linked to abdominal obesity, hyperglycemia, hypertension, hypertriglyceridemia in both genders. There were no specific gender differences. The total noodle intake had a higher association with insulin resistance in both genders, but significant differences were shown only in men. However, the total noodle intake was not significantly associated with serum hs-CRP, AST and ALT concentrations, and eGFR (Table 4).

### 3.4. A Causal Relationship between Total Noodle Intake with MetS and Its Components by a Two-Sample MR Analysis

Genetic variants linked to noodle intake were explored by conducting GWAS between low- and high-noodle intake groups according to the cutoff of 130 g/day noodle intake. The Q-Q plot in Supplementary Figure S1A showed the logarithm of the observed and expected *p*-values on the y-axis and x-axis, respectively. The inflation factor was 1.036, which suggested no inflation (Figure S1A). The Q-Q plot revealed that there were no biased genetic variants between the groups with low and high-noodle intake.

The *p*-values of genetic variants in the GWAS results are presented as a Manhattan plot in the Supplementary Figure S1B. Their associations were marginal since the intake of noodles is a lifestyle-related parameter. Furthermore, potential LD in the genetic variants was eliminated using an r^2^ threshold of < 0.001 within a distance of 10,000 kb using the R package. Therefore, the *p*-value cutoff for the GWAS between the categories of noodle intake was assigned as *p* < 5 × 10^−5^. The selected genetic variants from the GWAS that had a significant relationship with MetS and its components (*p* < 5 × 10^−5^) were removed. It resulted in 53 genetic variants that met the requirements for use as instrumental variables, the features of which are displayed in Supplementary Table S2.

Noodle intake with the cutoff of ≥130 g/day was only causally linked to a heightened risk of MetS in the IVW approach (OR = 1.196, 95% CI = 1.045~1.368, *p* = 0.0009) and not in the MR-Egger, weighted median, and weighted mode methods (*p* > 0.05; Table 3). Figure 2A revealed a positive correlation between a predicted MetS incidence from genetics and noodle intake, and the line passed zero in the IVW, MR-Egger, and weighted median methods. The slope of the lines, which represented the effect size of the connection between noodle intake and MetS risk, was equivalent in the IVW and MR-Egger methods (Figure 2A). Figure 2B showed the MR effect size for noodle intake on MetS in each instrumental variable. MetS was significantly associated with noodle intake in the overall instrumental variables in the IVW but not in the MR-Egger method (Figure 2B). Thus, the association in MR-Egger is similar to IVW, although MR-Egger was not significant. Since the MR-Egger method is known to be a reference for the direction of a causal and positive association in the two-sample MR analysis [28], the positive direction of the association was correctly represented. The slope in the weighted median was smaller than that in the IVW and MR-Egger.

**Figure 3:**
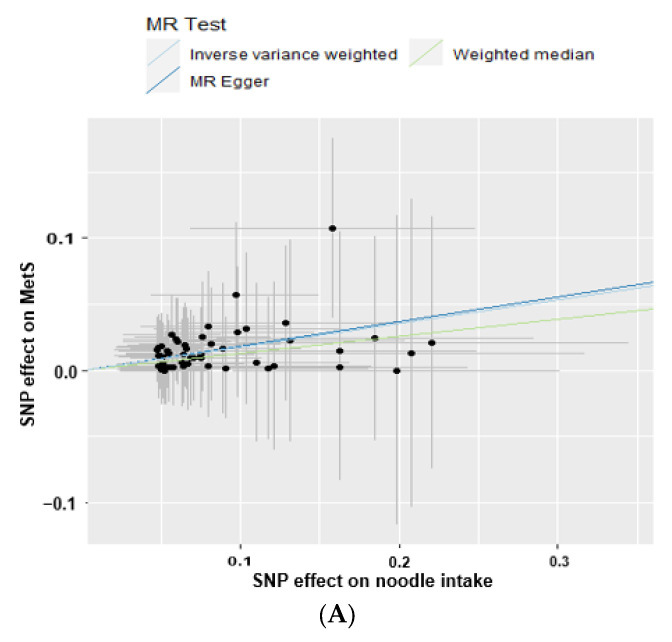

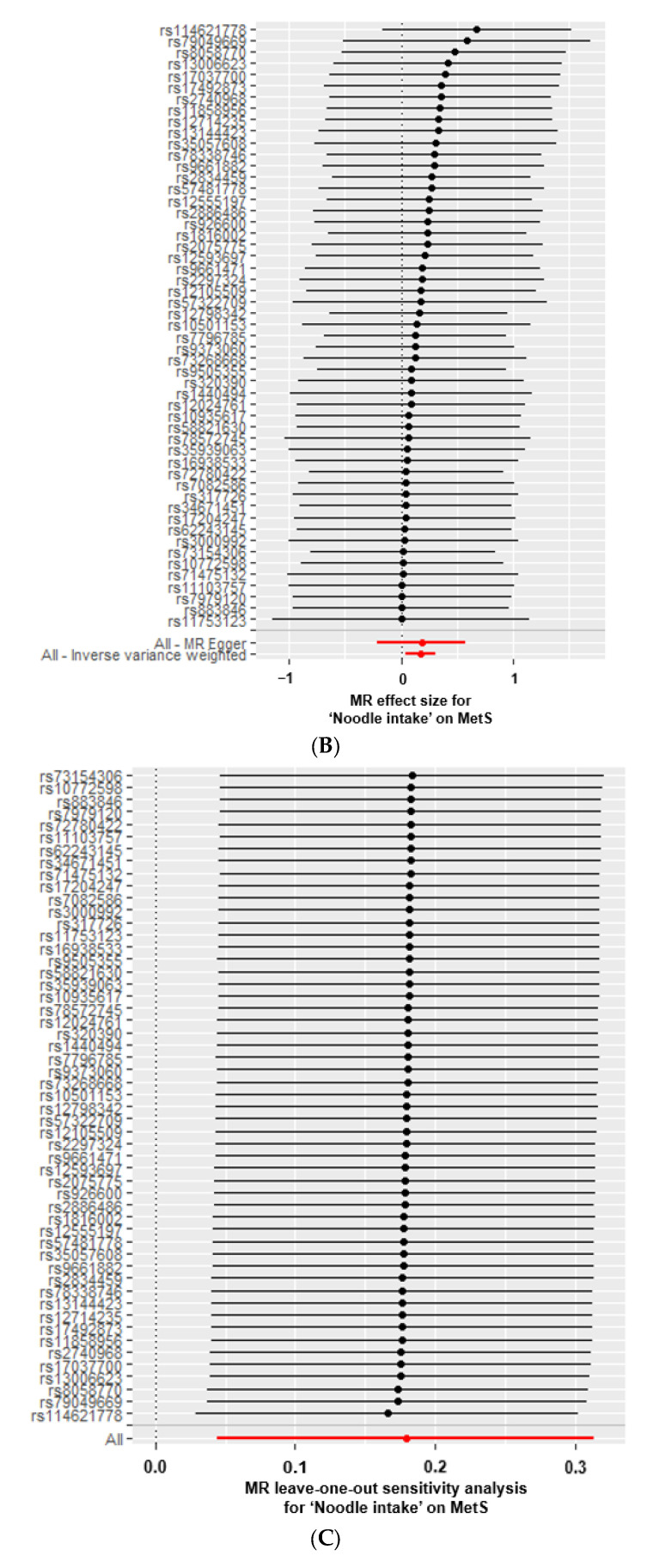

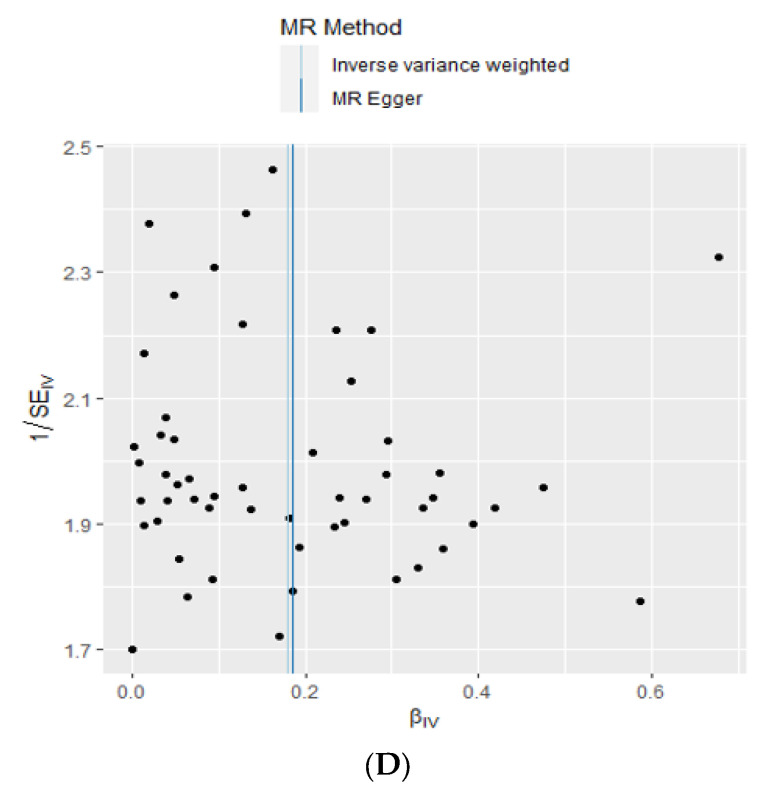
Two-sample Mendelian randomization (MR) analysis of the association between daily total noodle intake (cutoff: 130 g/day) and metabolic syndrome (MetS) risk. (**A**) Correlation between the effect size of the single nucleotide polymorphism (SNP)-total noodle intake (x-axis, standard deviation (SD) units) and SNP-MetS association (y-axis, log OR) to the SD bars. (**B**) Forest plot between daily total noodle intake and MetS. Each black dot represents an increase in the log OR of each SD in MetS, using each SNP of daily total noodle intake as a separate instrumental tool in the MR-Egger method. All–Egger and all-inverse-variance weighted (IVW) indicated a combined causal estimate using all SNPs in a single instrumental variable, using each MR-Egger and IVW random effects. The horizontal lines in each SNP represented the 95% confidence intervals. (**C**) Leave-one-out sensitivity analysis of MR for the effect of daily total noodle intake on the MetS. Each black dot indicated an IVW method for estimating the causal effect of daily total noodle intake on MetS, from which the single SNP was excluded. All described the IVW estimate using eight SNPs in the leave-one-out sensitivity analysis. (**D**) IVW and MR-Egger regression funnel plot for the effect of the daily total noodle intake on MetS. The vertical line represented a causal estimate using all SNPs combined into a single instrumental variable for each IVW and MR-Egger method. SNP, single nucleotide polymorphism; IVW, inverse-variance weighted.

Among the MetS components, the intake of noodles showed a positive association with hypertension and dyslipidemia only in the logistic regression analysis in the IVW method (*p* = 0.005 and *p* = 0.006, respectively; Table 3). In linear association, noodle intake was positively associated with serum triglycerides, LDL, hypo-HDL, and glucose concentrations only in the IVW method (*p* = 0.01, 0.02, 0.02, and 0.004, respectively) but not in the MR-Egger, weighted median, and weighted mode methods (*p* > 0.05; Table 3 and Figure 3A). Furthermore, body composition, BMI, waist circumferences, and fat mass exhibited a positive relationship with noodle intake only in the IVW method (*p* = 0.004, 0.01, and 0.01, respectively, Table 3). However, serum glucose concentrations, BMI, and waist circumferences were close to statistical significance in the weighted median method (*p* = 0.086, 0.064, 0.066, respectively). Since the weighted median estimator retained greater precision than the MR-Egger analysis, serum glucose concentrations, BMI, and waist circumferences were causally positively associated with noodle intake.

No heterogeneity was observed between noodle intake and MetS and its components in the MR-Egger and IVW analysis (*p* = 1; Table 3 and Figure 3B). The intercept of the association between noodle intake and the risk of MetS and its components was not significantly different from zero in the MR-Egger and IVW tests, demonstrating no significant horizontal pleiotropy of the association between them (*p* > 0.05; Table 3). Cochran’s Q test further indicated no heterogeneities in MetS and its components (all *p*-values of Cochran’s Q = 1). The exclusion of individual genetic variants in the leave-one-out analysis did not affect the association between total noodle intake and MetS and its components without removing one genetic variant by one (Figure 3C). Moreover, the lines for IVW and MR-Egger in the funnel plot for MetS were closely placed together. The distribution of genetic variants was close to symmetrical in the IVW and MR-Egger tests (Figure 3D). It indicates that the association between noodle intake and MetS did not manifest any signs of bias of the instrumental variables in the IVW and MR-Egger methods. The MR estimate of each SNP was plotted as a function of its MAF-corrected association with MetS. A MAF correction was used in proportion to the standard error of the SNP-MetS since a low MAF is likely to be measured with low accuracy. The plot revealed symmetry in the IVW and asymmetry in the MR-Egger method. Thus, noodle intake was causally and positively associated with MetS in the IVW method.

## 4. Discussion

Asians have a high grain intake from boiled grains or noodles. Noodles vary according to noodle sources, thickness, length, and shape. Noodles are mainly made of wheat and durum wheat, but rice, buckwheat, and potato starch are also used as raw materials to make noodles. Previous studies have demonstrated that noodle and pasta intake exhibit controversial results in MetS risk, possibly due to the differences in the sources and types of noodles [7,10]. Intake of instant noodles is shown to increase MetS risk in adults in the data from KNHANES IV 2007–2009 [7]. However, WHI in 84,555 postmenopausal women has shown that substituting pasta meals for other starchy foods could improve cardiometabolic outcomes [29]. In the INHES, pasta consumption was found to be inversely associated with the incidence of being overweight and obese [11]. However, few observational studies have been conducted on the relationship between the intake of noodles and MetS and its components. No study to date has been performed to identify their causal association. The present study revealed that not only the intake of instant noodles but also the intake of noodles overall was positively linked to MetS and waist circumference, hypo-HDL-cholesterolemia, hypertriglyceridemia, and hypertension. Furthermore, to our knowledge, overall noodle intake increased MetS incidence in a two-sample MR.

The association between noodle intake and MetS risk remains controversial. The differences in the relationship between noodle intake and MetS risk between Western countries and Asia are attributed to the differences in the dietary patterns between the two geographical regions. Consistent with the results of the present study, Asians having a high intake of noodles were also observed to consume a small amount of rice and a high amount of meat intake, including processed meats, as seen in two studies, one based on a Nutrition and Diet Investigation Project in Jiangsu Province, China [30] and the other from the China Health and Nutrition Survey (CHNS) [31]. Two studies have indicated that adults with WSD consume more wheat flour and meat and less rice, associated with an increased risk of MetS [31]. This present study confirmed the same results as the CHNS and revealed that WSD had a slight link with the risk of MetS. Additionally, when the consumption of noodles and meats was separated, it was apparent that noodles had a bigger influence on MetS than WSD. As a result, Koreans who often eat noodles are more vulnerable to MetS and its related components. More research needs to be conducted to understand the correlation between noodle consumption and the development of MetS in various populations in order to develop efficient interventions to reduce the MetS risk.

The relationship between noodle intake and MetS risk may be related to both GI and GL. Both parameters determine the postprandial glucose levels in insulin-sensitive and insulin-resistant individuals. GI is a measure of the quality of carbohydrates, and GL is the amount of glucose released based on the carbohydrate consumed [31,32,33]. GI is estimated based on increased serum glucose concentrations when a given amount of carbohydrates is consumed. Dietary GI, but not GL, was positively linked to MetS risk in a meta-analysis of observational studies, but the results remain inconsistent [31,34]. In a randomized clinical trial, serum glucose variability during 24 h decreased in the low-GI group compared to the high-GI group in people on a Mediterranean-style healthy eating pattern [32]. Noodles are reported to have a lower GI than rice, although this may vary according to the type of noodle or rice (GI: 48–52 for noodles and 50–93 for rice). The GI values of noodles (about 44–60), rice (70), and grains (41–60) are different depending on their gluten and amylose contents. We, therefore, used the GI values commonly listed in Korea [33]. The intake of noodles alone has lower GI and GL values than rice intake. However, the GI of mixed foods in a meal may affect MetS risk differently. The present study revealed that the daily food consumption of people in the high-noodle group had much higher total GI and GL values than those in the low-noodle group. This result could be due to more noodles being consumed in the meals of the former, with fewer vegetables, as opposed to a meal with rice, which includes soup, meat or a fish dish, two vegetable dishes, and kimchi. As such, this could explain the positive correlation between noodle intake and the risk of MetS, as the GI of the mixed food in a meal may affect the risk differently. In Korea, people who often consume meals composed mainly of noodles also consume a high-GI meal.

The current study was the first to demonstrate a causal connection between consuming noodles (at least three times a week and at least 130 g a day) and MetS and its components by utilizing a two-sample Mendelian randomization and a study of observation in people from Asia. In the present study, instrumental variables were generated with the GWAS between low- and high-noodle intake at a *p*-value of 5 × 10^−5^ after adjusting for covariates including age, gender, education, income, energy, carbohydrates, and alcohol consumption, smoking, and physical exercise. Those variables associated with MetS and its components were excluded from the instrumental variables. Finally, 53 genetic variants were selected as instrumental variables, and only six were at *p*-values of 5 × 10^−7^–5 × 10^−6^, and most were between 5 × 10^−5^ and 5 × 10^−6^. No study to date has explored the genetic variants related to noodle intake. However, the noodle-rich dietary pattern was observed to interact with the 22q13 loci, including patatin-like phospholipase domain-containing protein 3 (PNPLA3), which has been shown to influence non-alcoholic fatty liver risk in KoGES [34]. The genetic association of meat intake has been studied, but the genetic variants do not meet the Bonferroni statistical significance. However, rs7166776 in 15q26.1 is marginally significantly associated with total meat intake per 1000 kcal energy (*p* = 5.54 × 10^−8^). Furthermore, they are not genetically linked, and many genetic variants show LD relationships [35,36]. Therefore, loose statistical significance should be applied for selecting the instrumental variables associated with lifestyle-related parameters.

The instrumental variables selected for the MR were 53 genetic variants. IVW, weighted median, and MR-Egger are all methods used in two-sample MR studies that assess the causal relationship between exposures (noodle intake) and outcomes (MetS). The main difference between these three methods is their causal effect estimate. IVW uses a weighted average of all available genetic instruments to estimate the causal effect, while MR-Egger uses a linear regression model to assess the relationship between the genetic instrument and the outcome [28,37]. A weighted median of the available genetic instruments is also used to estimate the causal effect. IVW is often considered the gold standard in two-sample MR studies, as it has been shown to be more robust and reliable than MR-Egger in small sample sizes but to have more bias [38]. The present study showed a positive association of noodle intake with MetS and its components, including hypertension, dyslipidemia, hypertriglyceridemia, hypo-HDL concentrations, hyperglycemia, and abdominal obesity in the IVW but not the MR-Egger method. The results suggest that noodles may have a link to MetS and its components. Nonetheless, the MR-Egger and weighted median tests did not detect a significant association between noodle intake and MetS, implying that a larger sample size may be necessary to strengthen the statistical analysis. Further research would also be necessary to understand the relationship between them better.

The current study was novel as it demonstrated that noodle intake (≥130 g/day, ≥3 times/week) has a causal relationship with MetS and its components in Asians. The other advantage was using the large cohort data, which were conducted in a well-controlled manner, such as anthropometric, biochemical, and genotyping measurements. Usual food intake was determined with SQFFQ designed for Korean dietary patterns, and nutrient intake was calculated from the food intake using a computer-aided nutrition analysis program containing nutrient composition for Korean foods. However, this study has some limitations: First, the instrumental variables to explain the causal association between noodles and MetS risk included some bias since a loose statistical significance was applied [39]. Second, the two cohorts included 58,701 and 13,598 participants, which may not be sufficient to show statistical power in a two-sample MR study, although the participants were ethnically homogeneous. Third, SQFFQ can under- or overestimate the participants’ food intake since it contained only 106 food items and the portion size was 0.5, 1, or 1.5 of the one portion size. Finally, MetS-related environmental parameters were used as confounding factors. These included age, gender, education, income, smoking, physical exercise, energy, carbohydrates, and alcohol consumption. However, some factors could have been missed out, leading to bias. Therefore, the causal relationship between the intake of noodles and MetS may include a bias and requires further confirmation with a large randomized clinical trial.

## 5. Conclusions

Adults with a high intake of noodles (≥130 g/day, ≥3 times/week), regardless of the type of noodle, namely, wheat noodles, instant noodles, Chinese noodles, and buckwheat noodles, had a higher energy intake with poorer diet quality than those with a low-noodle intake in the KoGES. The intake of noodles, related to high GI, was positively associated with a 1.34-fold MetS risk. Furthermore, in a two-sample MR, the noodle intake exhibited a positive causal relationship with MetS and its components, including hypertension, dyslipidemia, hypertriglyceridemia, hypo-HDL concentration, hyperglycemia, and abdominal obesity in Asians who consume a high carbohydrate diet. Therefore, it is essential to recognize the potential health risks associated with the high consumption of noodles. Public health intervention strategies should be implemented to reduce the prevalence of MetS and its components by decreasing noodle consumption and/or consuming it with various quality foods, especially for Asians.

## Figures and Tables

**Table 1:** Demographic characteristics and lifestyles according to gender and noodle intake status.

	Men (*n* = 20,293)		Women (*n* = 38,408)	
	Low Intake of Noodles (*n* = 17,799)	High Intake of Noodle (*n* = 2494)	Low Intake of Noodles (*n* = 36,409)	High Intake of Noodle (*n* = 1999)
Age (years)	56.7 ± 0.06 ^a^	54.3 ± 0.16 ^b^	52.5 ± 0.04 ^c^	50.3 ± 0.179 ^d^***^+++^
Education (Yes, %) ≤Middle school High school ≥College	1554 (14.2) 8314 (75.8) 1105 (10.1)	199 (13.2) ^‡^ 1120 (74.4) 186 (12.4)	6529 (22.6) 20,711 (71.7) 1665 (5.76)	209 (14.1) ^‡‡‡^ 1160 (78.1) 117 (7.87)
Income (Yes, %) ≤$2000 $2000–4000 >$4000	1449 (8.57) 7179 (42.4) 8287 (49.0)	157 (6.55) ^‡‡^ 1028 (42.9) 1213 (50.6)	4038 (11.8) 15,144 (44.2) 15,049 (44.0)	134 (7.07) ^‡‡‡^ 841 (44.4) 921 (48.6)
Past smoker Smoker (Yes, %)	7795 (43.9) 4740 (26.7)	1000 (40.1) 924 (37.1) ^‡‡‡^	418 (1.15) 663 (1.83)	42 (2.11) 86 (4.31) ^‡‡‡^
Alcohol intake (g/day)	6.5 ± 0.11 ^b^	10.3 ± 0.28 ^a^	1.7 ± 0.08 ^c^	2.2 ± 0.31 ^c^***^+++##^
Physical activity (Yes, %)	10,547 (59.5)	1405 (56.4) ^‡‡^	19,091 (52.6)	933 (46.7) ^‡‡‡^
Energy (EER percent)	86.9 ± 0.24 ^d^	110.5 ± 0.61 ^b^	97.7 ± 0.16 ^c^	131.5 ± 0.66 ^a^***^+++###^
Carbohydrates (En%)	72.0 ± 0.06 ^a^	68.8 ± 0.14 ^b^	72.0 ± 0.04 ^a^	68.5 ± 0.15 ^b+++^
Protein (En%)	13.0 ± 0.02 ^d^	13.7 ± 0.05 ^b^	13.5 ± 0.01 ^c^	14.1 ± 0.06 ^a^***^+++^
Fat (En%)	13.7 ± 0.04 ^b^	16.5 ± 0.11 ^a^	13.6 ± 0.03 ^b^	16.5 ± 0.12 ^a+++^
Saturated fat (En%)	4.27 ± 0.01 ^c^	5.37 ± 0.03 ^a^	4.35 ± 0.01 ^b^	5.22 ± 0.03 ^a+++###^
Monounsaturated fat (En%)	5.46 ± 0.01 ^c^	6.86 ± 0.02 ^a^	5.35 ± 0.01 ^d^	6.72 ± 0.03 ^b^***^++^
Polyunsaturated fat (En%)	3.10 ± 0.02 ^b^	3.80 ± 0.04 ^a^	3.08 ± 0.01 ^b^	3.78 ± 0.05 ^a+++^
Cholesterol (mg/day)	162.7 ± 0.79 ^d^	193.3 ± 1.99 ^b^	168.0 ± 0.53 ^c^	210.1 ± 2.16 ^a^***^+++##^
Fiber (g/day)	16.1 ± 0.07 ^a^	16.4 ± 0.16 ^a^	14.0 ± 0.04 ^b^	13.1 ± 0.18 ^c^**
Calcium (mg/day)	453 ± 1.50 ^a^	373 ± 3.73 ^c^	449 ± 0.99 a	390 ± 5.1 ^b^***^+++###^
Sodium (g/day)	2.67 ± 0.01 ^b^	2.82 ± 0.02 ^a^	2.29 ± 0.01 ^c^	2.30 ± 0.03 ^c^***^+++##^
Potassium (g/day)	2.34 ± 0.01 ^a^	2.13 ± 0.01 ^c^	2.20 ± 0.003 ^b^	1.81 ± 0.02 ^d^***^+++###^
Vitamin C (mg/day)	105 ± 0.47 ^b^	87.3 ± 1.18 ^d^	109 ± 0.32 ^a^	77.3 ± 1.31 ^c^***^+++##^
Vitamin D (ug/day)	6.2 ± 0.04 ^b^	4.2 ± 0.09 ^c^	6.8 ± 0.02 ^a^	4.0 ± 0.10 ^c^*^+++###^
DII (scores)	−20.6 ± 0.12 ^a^	−18.6 ± 0.29 ^b^	−19.1 ± 0.08 ^b^	−15.0 ± 0.32 ^c^***^+++###^
Flavonoids (mg/day)	36.3 ± 0.25 ^b^	27.8 ± 0.62 ^c^	40.9 ± 0.16 ^a^	26.3 ± 0.68 ^c^**^+++###^

The values represent adjusted means ± standard deviations or the number of participants (percentage of each group). Covariates included age, residence areas, gender, body mass index, education, income, energy intake, carbohydrate intake, daily activity, and smoking status. EER, estimated energy requirement; CHO, carbohydrates; DII, dietary inflammatory index. A total noodle intake was the sum of instant noodles, wheat noodle soup, Chinese noodles (Chajangmyon/Jambbong), buckwheat noodles, and starch noodles daily. The cutoff of total noodle intake was 130 g/day, equivalent to three times a week. * Significant differences by gender at *p* < 0.05, ** at *p* < 0.01, *** *p* < 0.001. ^++^ Significant differences by noodle intake at *p* < 0.01, ^+++^ *p* < 0.001 ^##^ Significant effect of the interaction between gender and the noodle intake groups at *p* < 0.01, ^###^ *p* < 0.001. ^a,b,c,d^ Different superscript letters indicated significant differences among the groups in Tukey’s test at *p* < 0.05. ^‡^ Significant differences from the low-noodle intake in each gender at *p* < 0.05, ^‡‡^ at *p* < 0.01, and ^‡‡‡^ at *p* < 0.001.

**Table 2:** Usual food intake according to gender and noodle intake (unit: g/day).

	Men (*n* = 20,293)		Women (*n* = 38,408)	
	Low Intake of Noodles (*n* = 17,799)	High Intake of Noodle (*n* = 2494)	Low Intake of Noodles (*n* = 36,409)	High Intake of Noodle (*n* = 1999)
Total noodle	47.6 ± 0.49 ^c^	213.1 ± 1.22 ^b^	30.6 ± 0.32 ^d^	221.2 ± 1.35 ^a^***^+++###^
Instant noodles	10.8 ± 0.13 ^c^	30.9 ± 0.32 ^a^	5.21 ± 0.09 ^d^	22.7 ± 0.35 ^b^***^+++##^
Wheat noodle soup	18.3 ± 0.28 ^c^	85.3 ± 0.70 ^b^	15.6 ± 0.18 ^d^	108.5 ± 0.77 ^a^***^+++###^
Chinese noodle	14.7 ± 0.29 ^c^	84.3 ± 0.73 ^a^	7.1 ± 0.20 ^d^	74.7 ± 0.81 ^b^***^+++^
Buckwheat noodle	3.23 ± 0.08 ^c^	12.1 ± 0.20 ^b^	2.23 ± 0.05 ^d^	14.7 ± 0.22 ^a^***^+++###^
Starch noodle	0.56 ± 0.02 ^a^	0.57 ± 0.05 ^a^	0.43 ± 0.01 ^b^	0.54 ± 0.05 ^ab^*
White rice	147 ± 2.0 ^b^	179 ± 5.03 ^a^	82.6 ± 1.34 ^d^	113 ± 5.58 ^c^***^+++^
Whole grains	523 ± 2.04 ^a^	431 ± 5.07 ^c^	457 ± 1.35 ^b^	329 ± 5.62 ^d^***^+++###^
Bread	12.5 ± 0.19 ^c^	13.9 ± 0.46 ^b^	12.7 ± 0.12 ^c^	21.2 ± 0.51 ^a^***^+++###^
Cookie	3.07 ± 0.06 ^a^	2.44 ± 0.16 ^c^	2.72 ± 0.04 ^b^	2.53 ± 0.18 ^bc+++^
Potato	17.4 ± 0.21 ^b^	13.9 ± 0.52 ^c^	20.7 ± 0.14 ^a^	16.0 ± 0.57 ^b^***^+++^
Green vegetables	70.8 ± 0.52 ^a^	54.4 ± 1.30 ^c^	75.0 ± 0.35 ^b^	54.0 ± 1.47 ^c+++#^
White vegetables	45.9 ± 0.33 ^a^	39.6 ± 0.83 ^b^	41.0 ± 0.22 ^b^	30.6 ± 0.92 ^c^***^+++##^
Kimchi	158 ± 0.91 ^a^	143 ± 2.26 ^b^	131 ± 0.60 ^c^	103 ± 2.51 ^d^***^+++###^
Fruits	199 ± 1.68 ^b^	147 ± 4.19 ^c^	237 ± 1.11 ^a^	144 ± 4.64 ^c^***^+++###^
Beans	30.3 ± 0.20 ^a^	27.0 ± 0.50 ^c^	29.2 ± 0.13 ^b^	24.3 ± 0.55 ^d^***^+++#^
Seaweeds	1.81 ± 0.02 ^b^	1.40 ± 0.04 ^c^	2.12 ± 0.01 ^a^	1.55 ± 0.05 ^c^***^+++##^
Meats	47.7 ± 0.28 ^a^	44.3 ± 0.69 ^b^	33.8 ± 0.18 ^c^	25.8 ± 0.76 ^d^***^+++###^
Fish	35.5 ± 0.24 ^a^	26.3 ± 0.60 ^c^	33.5 ± 0.16 ^b^	20.5 ± 0.67 ^d^***^+++###^
Process meats	48.7 ± 0.72 ^a^	41.7 ± 1.79 ^b^	44.8 ± 0.48 ^b^	38.0 ± 1.99 ^bc^**^+++^
Milk and milk products	109.2 ± 1.04 ^b^	69.6 ± 2.60 ^c^	128.7 ± 0.69 ^a^	75.4 ± 2.88 ^c^***^+++##^
Nuts	1.6 ± 0.03 ^b^	1.1 ± 0.08 ^c^	1.9 ± 0.02 ^a^	1.3 ± 0.09 ^c^***^+++^
Coffee	4.2 ± 0.03 ^a^	4.2 ± 0.06 ^a^	3.3 ± 0.02 ^c^	3.5 ± 0.07 ^b^***^#^
Glycemic index	49.4 ± 0.10 ^c^	59.8 ± 0.28 ^a^	45.6 ± 0.08 ^d^	58.2 ± 0.32 ^b^***^+++###^
Glycemic load	154 ± 0.35 ^b^	193 ± 0.97 ^a^	142 ± 0.25 ^d^	186 ± 1.1576 ^b^***^+++###^
KBD (N, %)	6909 (38.8)	1278 (51.2) ^‡‡‡^	10,678 (29.3)	750 (37.5) ^‡‡‡^
PBD (N, %)	3719 (20.9)	563 (22.6) ^‡^	14,499 (39.8)	999 (50.0) ^‡‡‡^
WSD (N, %)	8044 (45.2)	2379 (95.4) ^‡‡‡^	11,389 (31.3)	1822 (91.2) ^‡‡‡^
RMD (N, %)	5577 (31.3)	947 (38.0) ^‡‡‡^	12,235 (33.9)	749 (37.5) ^‡‡^

The values are expressed as adjusted means ± standard deviations or the number of participants (percentage of each group). The covariates covered age, place of residence, gender, BMI, education, income, daily activity, energy consumption, carbohydrate consumption, and smoking status. A total noodle intake was the sum of instant noodles, wheat noodle soup, Chinese noodles (Chajangmyon/Jambbong), buckwheat noodles, and starch noodles daily. The cutoff of total noodle intake was 130 g/day, equivalent to three times a week. KBD, Korean-style balanced diet; PBD, plant-based diet; WSD, Western-style diet; RMD, rice-main diet. * Significant differences by gender at *p* < 0.05, ** *p* < 0.01, and *** *p* < 0.001. ^+++^ *p* < 0.001 ^#^ Significant effect of the interaction between gender and the noodle intake groups at *p* < 0.05, ^##^ *p* < 0.01, and ^###^ *p* < 0.001. ^a,b,c,d^ Different superscript letters indicated significant differences among the groups in Tukey’s test at *p* < 0.05. ^‡^ Significant differences from the low-noodle intake in each gender at *p* < 0.05, ^‡‡^ at *p* < 0.01, and ^‡‡‡^ at *p* < 0.001.

**Table 3:** Metabolic parameters related to metabolic syndrome (MetS) according to gender and noodle intake status.

	Men (*n* = 20,293)			Women (*n* = 38,408)		
	Low Intake of Noodles (*n* = 17,799)	High Intake of Noodles (*n* = 2494)	Adjusted OR	Low Intake of Noodles (*n* = 36,409)	High Intake of Noodle (*n* = 1999)	Adjusted OR
MetS (Yes, %) ^1^	3052 (17.2)	546 (21.9) ^‡‡‡^	1.341 (1.182–1.523)	4438 (12.2)	264 (13.2)	1.345 (1.144–1.580)
BMI (mg/kg^2^) ^2^	24.4 ± 0.02 ^b^	24.7 ± 0.07 ^a^	1.147 (1.045–1.260)	23.6 ± 0.02 ^c^	23.7 ± 0.08 ^c^***^+++^	1.165 (1.047–1.296)
Waist C. (cm) ^3^	84.3 ± 0.04 ^b^	84.7 ± 0.11 ^a^	1.263 (1.141–1.398)	78.8 ± 0.03 ^c^	79 ± 0.13 ^c^***^++#^	1.257 (1.111–1.423)
SMI (kg/m^2^) ^4^	7.2 ± 0.01 ^a^	7.3 ± 0.02 ^a^	1.047 (0.951–1.153)	6.1 ± 0 ^b^	6.1 ± 0.02 ^b^	1.068 (0.946–1.206)
Fat mass (%) ^5^	22.6 ± 0.01 ^c^	22.6 ± 0.02 ^b^	1.232 (1.115–1.361)	31.4 ± 0.01 ^a^	31.5 ± 0.03 ^a^***^++^	1.169 (1.055–1.295)
Plasma glucose (mg/dL) ^6^	98 ± 0.17 ^b^	100.1 ± 0.47 ^a^	1.210 (1.074–1.363)	93.5 ± 0.12 ^c^	94.5 ± 0.52 ^c^***^+++^	1.248 (1.053–1.479)
Blood HbA1 c (%) ^7^	5.67 ± 0.01 ^b^	5.73 ± 0.02 ^a^	1.297 (1.068–1.575)	5.73 ± 0.01 ^b^	5.73 ± 0.02 ^b#^	1.578 (1.226–2.031)
Insulin resistance (%) ^8^	1910 (11.1)	299 (14.5) ^‡‡‡^	1.288 (1.118–1.484)	1961 (6.04)	105 (6.65)	1.196 (0.970–1.475)
Serum total cholesterol (mg/dL) ^9^	190.6 ± 0.31 ^c^	193.2 ± 0.85 ^b^	1.202 (1.071–1.350)	201.3 ± 0.22 ^a^	200.3 ± 0.95 ^a+++##^	1.028 (0.915–1.155)
Serum HDL (mg/dL) ^10^	49.4 ± 0.11 ^b^	49.7 ± 0.3 ^b^	1.063 (0.945–1.195)	56.1 ± 0.08 ^a^	56.3 ± 0.33 ^a^***	1.071 (0.963–1.191)
Serum LDL (mg/dL) ^11^	113 ± 0.28 ^b^	112.8 ± 0.78 ^b^	1.137 (0.990–1.306)	122.1 ± 0.2 ^a^	119.9 ± 0.87 ^a^***	1.142 (1.001–1.302)
Serum TG (mg/dL) ^12^	140.9 ± 0.72 ^b^	153.4 ± 2 ^a^	1.199 (1.087–1.321)	116 ± 0.51 ^c^	120.8 ± 2.23 ^c^***^+++#^	1.284 (1.144–1.441)
SBP (mmHg) ^13^	124.6 ± 0.12 ^a^	125.1 ± 0.33 ^a^	1.097 (0.997–1.208)	121.3 ± 0.09 ^b^	121.2 ± 0.37 ^b^***	1.099 (0.981–1.230)
DBP (mmHg) ^14^	77.8 ± 0.08 ^a^	78.3 ± 0.22 ^a^	1.233 (1.079–1.409)	74.7 ± 0.06 ^b^	74.6 ± 0.25 ^b^***	0.971 (0.798–1.182)
Serum hs-CRP (mg/L) ^15^	0.142 ± 0.004	0.154 ± 0.01	1.013 (0.708–1.449)	0.136 ± 0.003	0.148 ± 0.011	1.093 (0.696–1.716)
Serum urate (mg/dL) ^16^	5.54 ± 0.01	5.62 ± 0.02	1.126 (1.023–1.240)	4.22 ± 0.01	4.25 ± 0.02	1.360 (1.065–1.736)
eGFR (ml/min) ^17^	84.7 ± 0.13 ^b^	84.6 ± 0.33 ^b^	1.273 (0.996–1.628)	86.5 ± 0.09 ^a^	86.7 ± 0.36 ^a^***	1.288 (0.962–1.724)
Serum AST (U/L) ^18^	24.7 ± 0.24 ^a^	26 ± 0.59 ^a^	1.169 (0.972–1.407)	23.1 ± 0.16 ^b^	23.1 ± 0.64 ^b^***	1.025 (0.790–1.329)
Serum ALT (U/L) ^19^	25.5 ± 0.23 ^b^	27.2 ± 0.56 ^a^	1.065 (0.945–1.201)	20.7 ± 0.16 ^c^	20.4 ± 0.61 ^a^***^+++##^	0.905 (0.751–1.092)

Values represent adjusted mean ± standard errors and adjusted odds ratio (OR) with 95% confidence intervals (CI). Adjusted OR was calculated with low-noodle intake as the reference using logistic regression with the adjustment of covariates and the cutoff points of each variable as following: ^1^ MetS criteria, ^2^ <25 kg/m^2^ for body mass index (BMI); ^3^ < 90 cm for men and 85 cm for women waist circumferences (C.); ^4^ <7.4 kg/m^2^ for men, 6.2 kg/m^2^ for women skeletal mass index (SMI) calculated with dividing skeletal muscle mass by squares of height; ^5^ <25% for men and 30% for women fat mass; ^6^ <126 mL/dL fasting serum glucose plus diabetic drug intake; ^7^ <6.5% hemoglobin A1c (HbA1c) plus diabetic drug intake; ^8^ <HOMA-IR for insulin resistance; ^9^ <230 mg/dL serum total cholesterol (chol) concentrations; ^10^ >40 mg/dL for men and 50 mg/dL for women serum high density lipoprotein (HDL) cholesterol; ^11^ <160 mg/dL low-density lipoprotein (LDL) concentrations; ^12^ <150 mg/dL plasma triglyceride (TG) concentrations; ^13^ <140 mmHg systolic blood pressure (SBP), ^14^ < 90 mmHg diastolic blood pressure (DBP) plus hypertension medication; ^15^ <0.5 mg/dL serum high-sensitive C-reactive protein (hs-CRP) concentrations; ^16^ <6 mg/dL Uric acid; ^17^ Estimated glomerular filtration rate (eGFR) <60; ^18^ Aspartate aminotransferase (AST) <40 U/L; ^19^ Alanine aminotransferase (ALT) <35 U/L. CI, 95% confidence intervals. A total noodle intake was the sum of instant noodles, wheat noodle soup, Chinese noodles (Chajangmyon/Jambbong), buckwheat noodles, and starch noodles daily. The cutoff of total noodle intake was 130 g/day, equivalent to three times a week. *** Significant differences by gender at *p* < 0.001. ^++^ Significant differences by noodle intake at *p* < 0.01 and ^+++^ *p* < 0.001 ^#^ Significant the interaction of gender and the noodle intake groups at *p* < 0.05 and ^##^ *p* < 0.01. ^a,b,c,d^ Different superscript letters indicated significant differences among the groups in Tukey’s test at *p* < 0.05. ^‡‡‡^ Significant differences from the low-noodle intake in each gender at ^‡‡‡^ at *p* < 0.001.

**Table 4:** Association of noodle intake with metabolic syndrome and its components using genetic variant randomization in different MR methods.

9	MR			Heterogeneity			Pleiotropy		
	Method	OR (95% CI)	*p*	Method	Q	*p*-Value	Intercept	SE	*p*-Value
Metabolic syndrome	MR-Egger	1.204(0.811~1.787)	0.362	MR-Egger	5.214	1	−0.0005	0.014	0.973
	WMD ^a^	1.137(0.957~1.352)	0.145						
	IVW	1.196(1.045~1.368)	0.0009	IVW	5.215	1			
	WMO ^b^	1.061(0.740~1.521)	0.748						
Hypertension	MR-Egger	1.089(0.858~1.383)	0.486	MR-Egger	4.436	1	0.002	0.008	0.787
	WMD ^a^	1.097(0.984~1.222)	0.095						
	IVW	1.124(1.036~1.218)	0.005	IVW	4.510	1			
	WMO ^b^	1.095(0.872~1.375)	0.437						
Dyslipidemia	MR-Egger	1.236(0.833~1.835)	0.298	MR-Egger	3.878	1	−0.002	0.014	0.899
	WMD ^a^	1.151(0.966~1.373)	0.116						
	IVW	1.206(1.055~1.379)	0.006	IVW	3.894	1			
	WMO ^b^	1.105(0.743~1.644)	0.624						
Exposures	Method	β (95% CI)	*p*	Method	Q	*p*-value	Intercept	SE	*p*-value
Serum triglyceride concentrations (mg/dL)	MR-Egger	0.145(−0.181~0.471)	0.387	MR-Egger	2.285	1	−0.000089	0.011	0.994
	WMD ^a^	0.117(−0.022~0.257)	0.100						
	IVW	0.144(0.033~0.255)	0.011	IVW	2.285	1			
	WMO ^b^	0.103(−0.185~0.392)	0.486						
Serum LDL concentrations (mg/dL)	MR-Egger	0.119(−0.210~0.447)	0.482	MR-Egger	3.301	1	0.0007	0.011	0.949
	WMD ^a^	0.107(−0.034~0.248)	0.138						
	IVW	0.129(0.017~0.240)	0.024	IVW	3.306	1			
	WMO ^b^	0.049(−0.249~0.346)	0.750						
Serum HDL concentrations (mg/dL)	MR-Egger	0.121(−0.105~0.346)	0.300	MR-Egger	3.081	1	−0.002	0.008	0.788
	WMD ^a^	0.080(−0.013~0.173)	0.091						
	IVW	0.091(0.015~0.168)	0.020	IVW	3.154	1			
	WMO ^b^	0.061(−0.147~0.269)	0.569						
Serum glucose concentrations (mg/dL)	MR-Egger	0.282(−0.084~0.648)	0.138	MR-Egger	5.709	1	−0.007	0.013	0.580
	WMD ^a^	0.141(−0.020~0.303)	0.086						
	IVW	0.184(0.060~0.308)	0.004	IVW	6.019	1			
	WMO ^b^	0.074(−0.237~0.386)	0.642						
BMI	MR-Egger	0.097(−0.119~0.313)	0.384	MR-Egger	3.729	1	0.0007	0.007	0.922
	WMD ^a^	0.087(−0.005~0.180)	0.064						
	IVW	0.107(0.034~0.180)	0.004	IVW	3.739	1			
	WMO ^b^	0.068(−0.133~0.269)	0.511						
Waist circumferences (cm)	MR-Egger	0.208(−0.243~0.659)	0.370	MR-Egger	2.479	1	−0.001	0.016	0.945
	WMD ^a^	0.179(−0.012~0.370)	0.066						
	IVW	0.193(0.039~0.347)	0.014	IVW	2.484	1			
	WMO ^b^	0.136(−0.240~0.513)	0.481						
Body fat	MR-Egger	0.218(−0.222~0.658)	0.337	MR-Egger	4.117	1	−0.002	0.015	0.889
	WMD ^a^	0.151(−0.047~0.349)	0.134						
	IVW	0.188(0.038~0.338)	0.014	IVW	4.136	1			
	WMO ^b^	0.100(−0.303~0.502)	0.630						

MR, Mendelian randomization; WMD ^a^, Weighted median; IVW, inverse-variance weighted mode. WMO ^b^, Weighted mode.

## Data Availability

Data can be obtained from Korea Biobank.

## Supplementary Materials

The following supporting information can be downloaded at: https://www.mdpi.com/article/10.3390/nu15092091/s1. Table S1: The factor-loading values of the predefined 29 food groups in each dietary pattern. Table S2: Instrumental variables for metabolic syndrome according to total noodle intake. Figure S1: Manhattan and quantile-quantile (QQ) plots for single nucleotide polymorphism (SNP) association with noodle intake.
