# Supplementary material for: A Positive Causal Relationship between Noodle Intake and Metabolic Syndrome: A Two-Sample Mendelian Randomization Study
Source: Nutrients. 2023 Apr 26;15(9):2091. doi: 10.3390/nu15092091 (PMC10181479; doi:10.3390/nu15092091)
Supplement: Supplementary file 1 [file nutrients-15-02091-s001.zip › nutrients-2361728-supplementary.pdf]

Table S1. Factor-loading values of the predefined 29 food groups in each dietary pattern.

	Korean balanced diet		Plant-based diet		Western-style diet		Rice-main diet	
Rice	-3		-7		4		93	*
Whole grain	9		-4		-2		-93	*
Noodles	2		2		62	*	3	
Bread	-7		35		53	*	-5	
Cookies	-7		31		30		6	
Beans	32		48	*	3		2	
Potatoes	24		50	*	5		-3	
Kimchi	50	*	1		-1		-3	
Eggs	8		43	*	15		5	
Fast food	-4		15		76	*	-4	
Green vegetables	67	*	42	*	-2		-2	
White vegetables	70	*	29		2		2	
Mushrooms	49	*	35		-6		-4	
Fatty fish	53	*	23		11		0	
White fish	66	*	17		12		0	
Crab	49	*	4		19		1	
Processed meat	18		15		6		-1	
Red meat	46	*	-7		41	*	8	
Chicken	33		-6		39		4	
Soup	16		4		65	*	-5	
Seaweed	44	*	41	*	-2		-4	
Milk	11		49	*	2		0	
Beverages	20		32		6		2	
Coffee	10		-1		19		15	
Tea	14		-8		24		14	
Fruit	19		48	*	-6		-6	
Pickles	50	*	-1		4		2	
Alcohol	18		-28		16		6	
Nuts	-2		50	*	6		-5	
Variance explained by each factor	3.545		2.487		2.291		1.799	

Values are factor-loading values, and their absolute values are higher than 0.4, indicating the principal component of each diet. The principal components of the food groups are flagged by an '*', and the name of each dietary pattern was assigned from the flagged principal food groups.

Table S2. Instrumental variables for metabolic syndrome according to total noodle intake.

SNP	CHR	Gene name	Effect allele	Other allele	Beta value	Standard error	P value for beta
rs9661471	1	Unknown	C	T	0.064458	0.03623	4.24E-05
rs3000992	1	Unknown	G	A	0.120903	0.06244	8.16E-06
rs12024761	1	Unknown	A	G	0.063333	0.03471	2.55E-05
rs320390	1	RPS6KC1	A	G	0.051153	0.02798	2.64E-05
rs9661882	1	ENSG00000235152	C	T	0.097951	0.04907	4.16E-06
rs72780422	2	LINC01250	T	C	-0.06419	0.03138	2.46E-06
rs12714235	2	PLB1	T	G	-0.04735	0.02673	4.51E-05
rs17037700	2	ENSG00000282890	G	C	0.059185	0.03306	3.74E-05
rs17204247	2	ENSG00000237844	G	A	0.058046	0.03108	1.61E-05
rs57481778	2	ENSG00000237844	G	C	0.053846	0.0297	3.17E-05
rs12105509	2	ENSG00000287621	A	G	0.089198	0.04762	1.58E-05
rs13006623	2	KLF7	A	G	0.079543	0.04173	1.09E-05
rs883846	3	CNTN4	A	G	0.051924	0.02747	1.39E-05
rs62243145	3	RBMS3	C	A	-0.05291	0.02838	1.77E-05
rs1440494	3	RBMS3	C	T	0.162266	0.08682	1.68E-05
rs10935617	3	Unknown	A	G	0.067071	0.03617	1.87E-05
rs17492873	3	ENSG00000289524	A	G	0.060698	0.03379	3.49E-05
rs73154306	3	RSRC1	T	C	-0.09135	0.04435	2.12E-06
rs2886486	3	Unknown	T	C	0.081347	0.0444	2.36E-05
rs57322709	4	ENSG00000251488	T	A	0.130977	0.0733	3.89E-05
rs13144423	4	LOC105377565	G	C	0.076276	0.04274	3.93E-05
rs34671451	4	Unknown	G	A	-0.07998	0.04434	3.29E-05
rs78572745	5	HSD17B4	A	G	0.207634	0.11	1.37E-05
rs9505355	6	Unknown	G	A	-0.22069	0.1238	4.03E-05
rs11753123	6	Unknown	T	A	0.198932	0.103	8.65E-06
rs9373060	6	TARID	G	T	-0.07068	0.03619	6.87E-06
rs317726	7	CPVL	T	C	-0.0535	0.0303	4.75E-05
rs7796785	7	CD36	G	A	-0.18509	0.09995	2.02E-05
rs2740968	8	CSMD1	G	T	0.05038	0.0274	2.14E-05
rs114621778	8	POP1	C	T	-0.15833	0.08951	4.66E-05
rs2297324	9	GLIS3	G	A	0.089198	0.04988	3.77E-05
rs16938533	9	Unknown	A	T	0.05038	0.02753	2.55E-05
rs12555197	9	FRMD3	G	A	-0.06596	0.03501	1.45E-05
rs11103757	9	ENSG00000227958	G	A	0.05038	0.02772	3.05E-05
rs73268668	10	Unknown	T	A	0.074816	0.03927	1.19E-05
rs1816002	10	ADAMTS14	T	C	0.054613	0.02657	2.40E-06
rs7082586	10	ADAMTS14	A	G	0.054613	0.02856	1.08E-05

rs12798342	11	PSMA1	A	T	-0.0751	0.03438	4.93E-07
rs10501153	11	PRR5L	T	C	0.068928	0.03774	2.71E-05
rs71475132	11	Unknown	A	G	0.162564	0.08095	3.73E-06
rs10772598	12	APOLD1	G	A	-0.11719	0.06392	2.44E-05
rs7979120	12	Unknown	C	T	-0.05081	0.0278	2.58E-05
rs35057608	15	CHRNA7	T	G	0.104146	0.05875	4.55E-05
rs11858956	15	Unknown	T	C	-0.049	0.02753	4.14E-05
rs12593697	15	FAM169B	C	G	-0.05134	0.02786	2.21E-05
rs58821630	16	ZFHX3	A	G	0.048442	0.02668	2.97E-05
rs8058770	16	KIAA0513	C	A	0.057286	0.03057	1.68E-05
rs78338746	17	LOC105371508	C	G	-0.06505	0.0358	2.87E-05
rs79049669	18	LOC107985176	C	G	0.09691	0.05307	2.68E-05
rs926600	20	Unknown	G	A	-0.04842	0.02722	4.19E-05
rs2075775	20	OGFR	T	C	0.049606	0.02822	4.85E-05
rs2834459	21	ENSG00000272657	C	T	-0.12889	0.06958	1.99E-05
rs35939063	21	BACE2	G	A	0.110253	0.05899	1.64E-05

CHR, chromosome.

Figure S1. Manhattan and quantile-quantile (QQ) plots for single nucleotide polymorphism (SNP) association with noodle intake

A. Manhattan plot

B. QQ plot

Daily total noodle intake was calculated by summing the daily intakes of instant noodle, noodle soup, Chinese noodle, buckwheat noodle, and starch noodle. The cutoff of total noodle was 130 g/day. Genetic variants of the noodle intake were determined with a genome-wide association study (GWAS).

A.

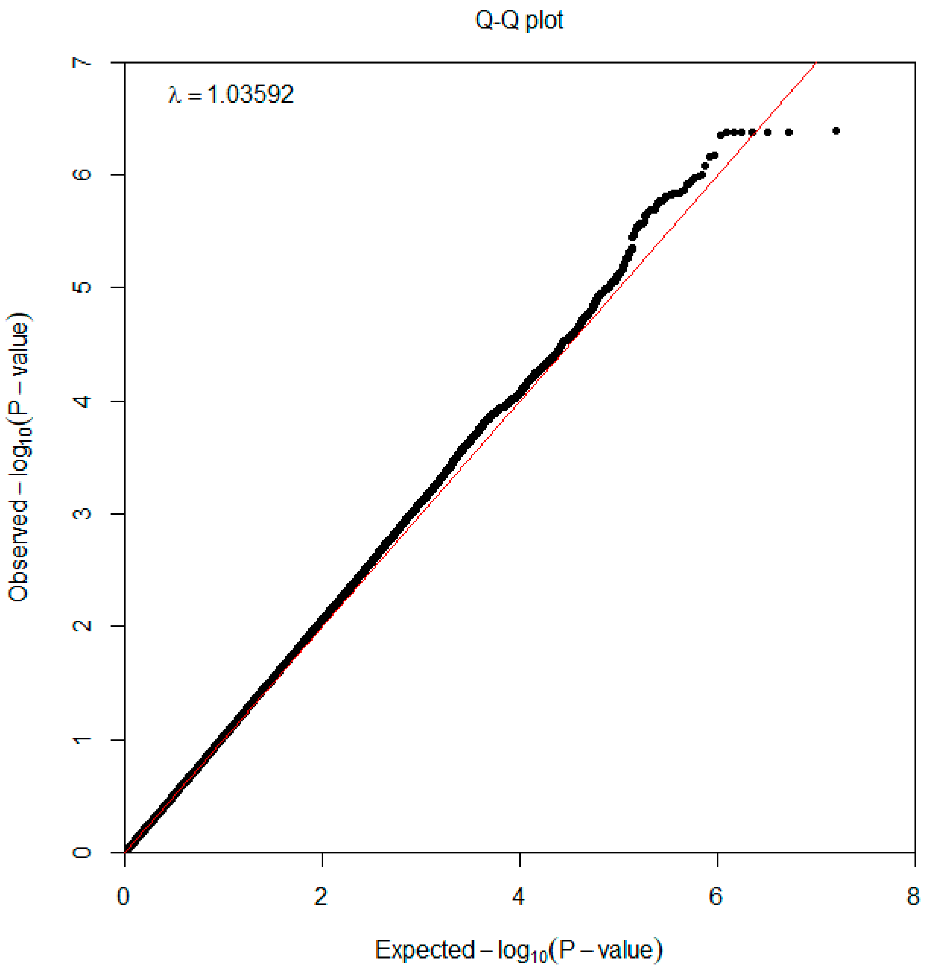

B.

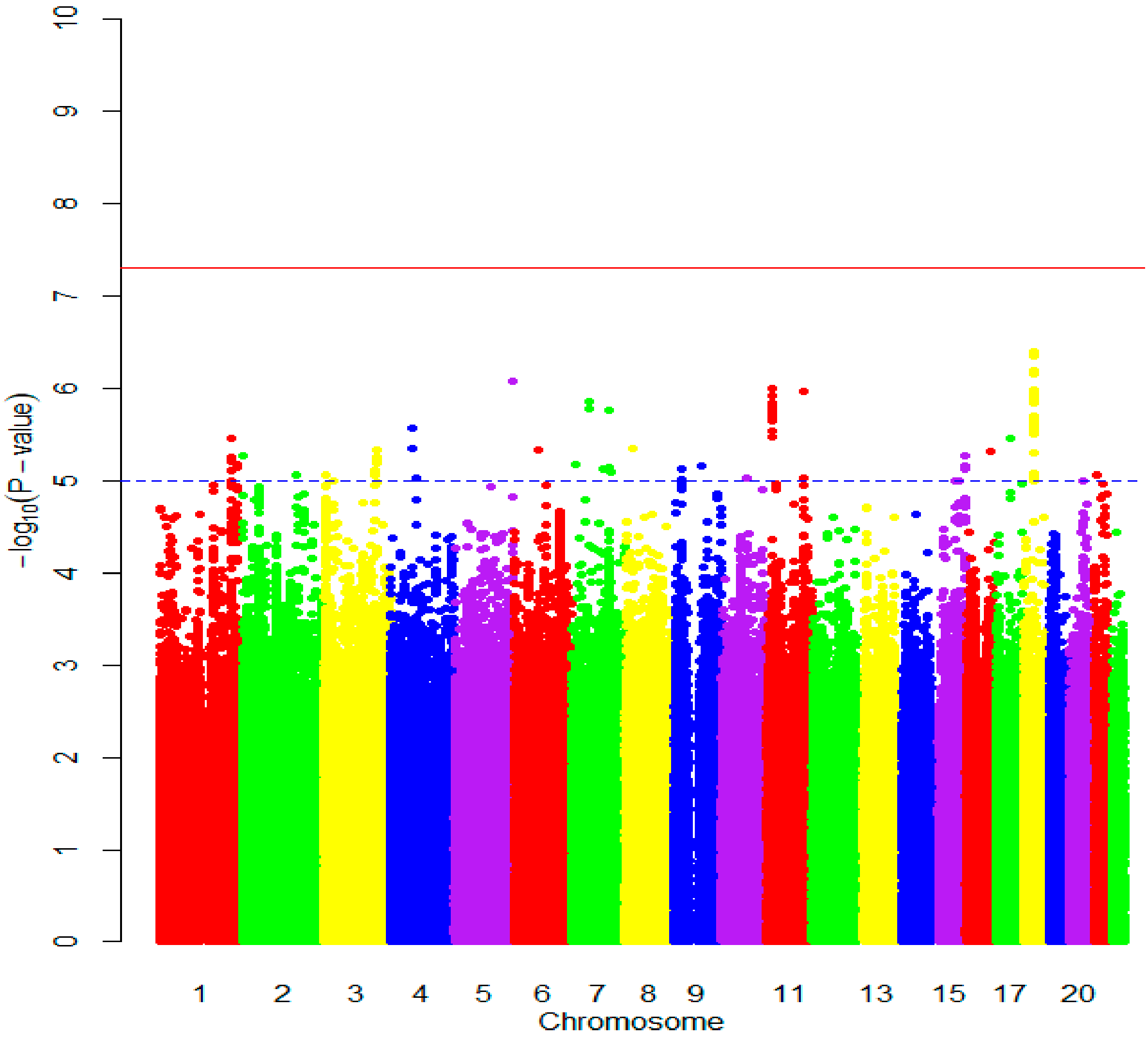

-log10(P-value)
0
1
2
3
4
5
6
7
8
9
10
1
2
3
4
5
6
7
8
9
11
13
15
17
20
Chromosome
